# Primary Neuroendocrine Tumors of the Breast: A Case Report and Literature Review

**DOI:** 10.7759/cureus.107894

**Published:** 2026-04-28

**Authors:** Despoina Milonaki, Nikoleta Sinou, Natalia Sinou, Dimitrios G Armamentos, Ioannis Provatas

**Affiliations:** 1 General Surgery, Agios Panteleimon General Hospital of Nikaia, Athens, GRC; 2 School of Medicine, Research and Education Institute in Biomedical Sciences, National and Kapodistrian University of Athens, Athens, GRC; 3 Anatomy, School of Medicine, National and Kapodistrian University of Athens, Athens, GRC; 4 Pathology, Evangelismos General Hospital, Athens, GRC

**Keywords:** breast, lumpectomy, neuroendocrine, pathology, primary

## Abstract

Primary neuroendocrine tumors (NETs) of the breast are a very rare type of tumor, originating from cells that make up the neuroendocrine system and produce peptides and amines. This type of tumor represents a very rare subtype of breast carcinoma, occurring mainly in postmenopausal women. Breast NETs are graded as well-differentiated G1, moderately differentiated G2, and poorly differentiated G3 (small or large cell neuroendocrine carcinoma), according to the Nottingham system. Neuroendocrine carcinomas usually affect the gastrointestinal and the pulmonary system.

We present a rare case of an 88-year-old woman who presented with a right breast mass, which was identified 1.5 months prior. The patient reported mild pain and localized tenderness throughout the day. She underwent a right breast lumpectomy for a palpable mass located in the upper inner quadrant. The pathological identification revealed histologic sections of neoplastic mammary gland tissue exhibiting neuroendocrine immunomorphological features, consistent with a well-differentiated grade. This case highlights the rarity of primary neuroendocrine tumors of the breast and the importance of an early diagnosis. Further studies are needed in order to better understand the biological behavior of the tumor and discover optimal therapies to maximize patient outcomes.

## Introduction

Neuroendocrine neoplasms (NENs) are a heterogeneous group of epithelial tumors characterized by morphological and immunohistochemical evidence of neuroendocrine differentiation [1]. They originate from the diffuse endocrine system, and their behavior depends on the differentiation of the tumor. They can occur in almost every organ system, most commonly the gastroenteropancreatic (GEP) and bronchopulmonary systems. Recent epidemiological data suggest they represent less than 0.1% of all breast malignancies and approximately 1% of all neuroendocrine tumors, making them one of the rarest subtypes of breast cancer [1-4].

The classification of these tumors has changed significantly over time. In the past, they were described as a “carcinoid” growth pattern based only on how they looked under the microscope. In 1977, eight additional cases with similar patterns were reported [2,3]. Breast neuroendocrine carcinoma (NEC) was first recognized as a distinct entity in the 2003 WHO Classification of Breast Tumors. The term was later refined in 2012 and subsequently harmonized with the general framework for NENs in the 2019 fifth edition and 2022 “Blue Book.” According to the WHO classification, NENs can be stratified based on their histological differentiation into - (a) well-differentiated (low grade) (G1), intermediate (G2) (neuroendocrine tumor {NET}), where G1 and G2 have a low proliferation index Ki-67, and high grade (G3); (b) poorly differentiated (small or large cells NEC) that have a high proliferation index Ki-67 and, typically, a very aggressive behavior; and (c) invasive breast carcinomas of no special type (IBC-NST) with neuroendocrine features. The Nottingham system is a method for grading breast cancer by evaluating its aggressiveness based on the following three factors: the degree of tubule formation, nuclear pleomorphism, and mitotic count. The total score determines the final grade, with a total of eight or nine being grade 3 (poorly differentiated), six or seven being grade 2 (moderately differentiated), and three to five being grade 1 (well-differentiated). This grade is different from the cancer's stage and helps doctors assess prognosis and treatment options. NENs (G1-G2) are often somatostatin receptor (SSTR)-positive, whereas NECs are high-grade and SSTR-negative, behaving more like small-cell or large-cell carcinomas, with rapid progression and poor prognosis [2].

Clinically, distinguishing primary breast NETs from metastatic neuroendocrine disease is paramount. Primary cases typically occur in postmenopausal women, express neuroendocrine markers like chromogranin A and synaptophysin in >50% of cells, and, unlike many extra-mammary NENs, often demonstrate estrogen (ER) and progesterone (PR) receptor positivity [2,5].

There are no standardized guidelines for the diagnosis and treatment of breast NETs, due to their rarity. The treatment is based on breast cancer protocols as follows: surgery for localized disease, adjuvant endocrine therapy if they are ER+/PR+, and chemotherapy for high-grade NECs. For aggressive NEC-like cases, a platinum-based chemotherapy regimen is used, similarly to small-cell carcinoma [2,5].

This report highlights a rare case of a primary G2 ΝΕΤ of the breast in an 88-year-old female. The aim of this report is to highlight and analyze a rare case of a primary NET of the breast, providing detailed macroscopic, microscopic, and immunohistochemical findings and the management of elderly patients.

## Case presentation

An 88-year-old woman with a medical history of hypertension and chronic venous insufficiency presented to the outpatient breast surgery clinic in November 2022 for evaluation of a right breast mass. She reported no known allergies, was a non-smoker, and did not consume alcohol. She had been vaccinated against COVID-19. At initial evaluation, the patient reported mild intermittent discomfort in the right breast. Clinical examination revealed no palpable mass or skin changes.

**Figure 1:**
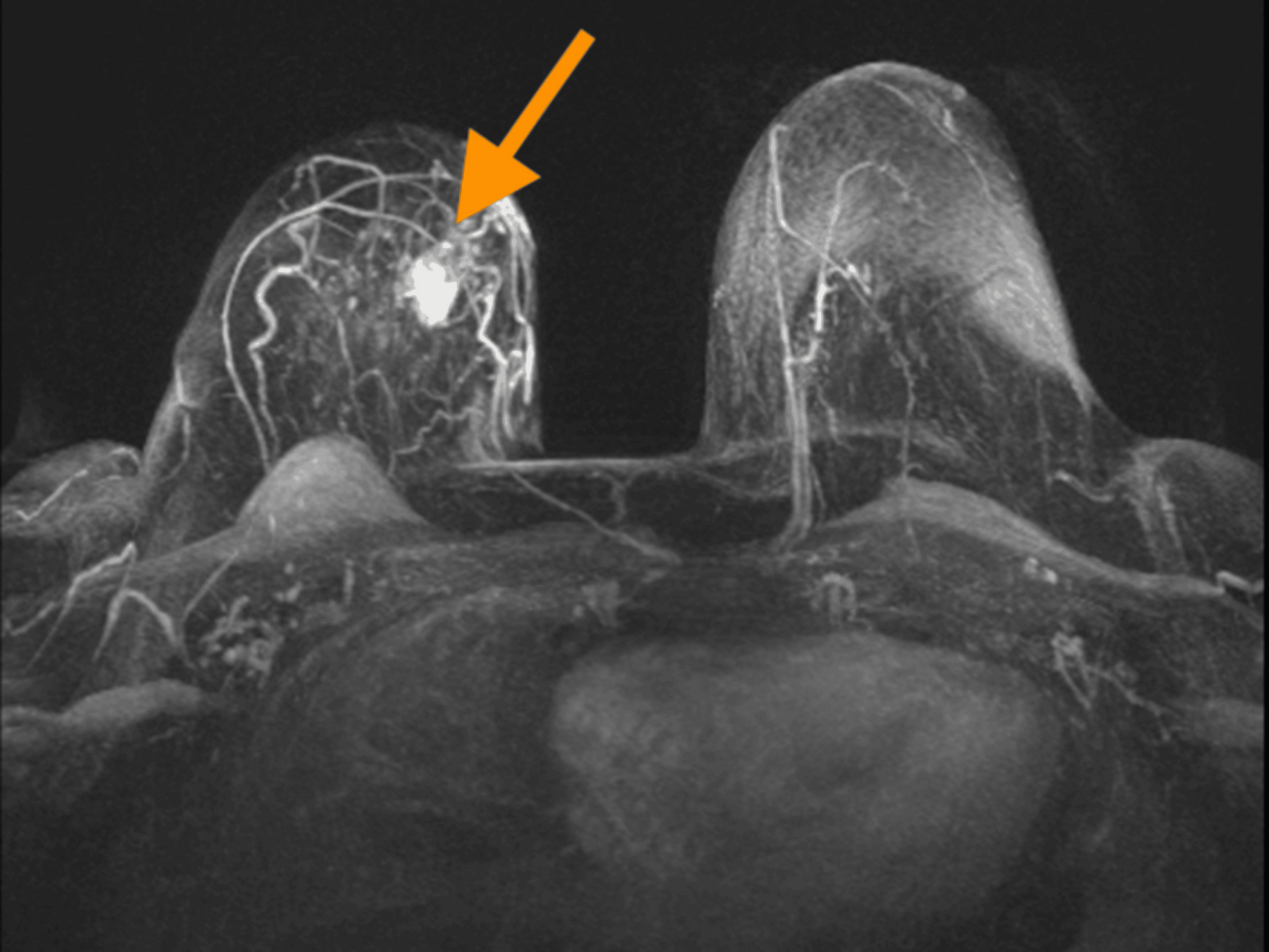
Postcontrast image demonstrating a mass with irregular rim enhancement (arrow) in the lateral position of the right breast, corresponding to the known malignant lesion.

A comprehensive imaging workup was performed. Digital mammography (November 2022) demonstrated a 1.5×1.0 cm lesion with ill-defined margins and a lobulated contour, located centrally in the right breast, slightly medial to the midline. Breast MRI revealed a lobulated lesion measuring 1.9×1.81×1.53 cm at the 2 o’clock position of the right breast, approximately 3.2 cm from the nipple and 3.7 cm anterior to the pectoralis major muscle. No additional focal lesions or abnormal enhancement were observed bilaterally (Figure 1).

Breast ultrasound identified a 1.3×1.2 cm hypoechoic mass in the upper inner quadrant of the right breast with irregular margins, non-parallel orientation (taller-than-wide), posterior acoustic shadowing, and internal vascularity on Doppler imaging. Ipsilateral axillary lymph nodes showed mild cortical thickening without loss of the fatty hilum. The lesion was classified as Breast Imaging Reporting and Data System (BI-RADS) 4C.

Histological analysis of core-needle biopsy specimens revealed a well-differentiated neuroendocrine tumor with predominantly solid architecture, low mitotic activity, and no evidence of necrosis. Immunohistochemistry demonstrated strong estrogen and progesterone receptor positivity (~95%), Ki-67 <5%, and HER2 negativity (1+).

Preoperative staging included whole-body computed tomography, bone scintigraphy, and laboratory evaluation, including tumor markers, all of which were negative for an extra-mammary primary tumor or metastatic disease. Additionally, preoperative cardiological and pulmonological assessments were unremarkable.

Following multidisciplinary discussion and completion of the diagnostic workup, the patient was informed about the available surgical options, including mastectomy, recommended in light of her overall condition, and breast-conserving surgery followed by radiotherapy. The patient opted for breast-conserving surgery. Preoperatively, she declined a sentinel lymph node biopsy.

In December 2022, the patient underwent a right breast lumpectomy under general anesthesia. The lesion was located in the upper inner quadrant, approximately 1.5 cm from the nipple, extending from the 1 to 3 o’clock position, measuring approximately 2×1.5 cm. A para-areolar incision was performed, followed by careful dissection through the skin and subcutaneous tissue down to the pectoralis major fascia. The tumor was excised with clear surgical margins, and the specimen was submitted for histopathological examination. Postoperatively, the patient declined further oncological treatment, including adjuvant radiotherapy and chemotherapy.

Histopathological findings

The right breast specimen, weighing 59 g and measuring 7×5×3 cm, included a spindle-shaped section of skin (5.3×1.8 cm) and contained a 1.7×1.5×1.4 cm irregular tumor, located 1.5 cm from the skin, 2 cm from the superior clip, 1 cm from the medial clips, and 3.5 cm from the lateral clips. The remaining parenchyma showed areas of fibrosis and atrophy of duct-lobular units (microscopically).

**Figure 2:**
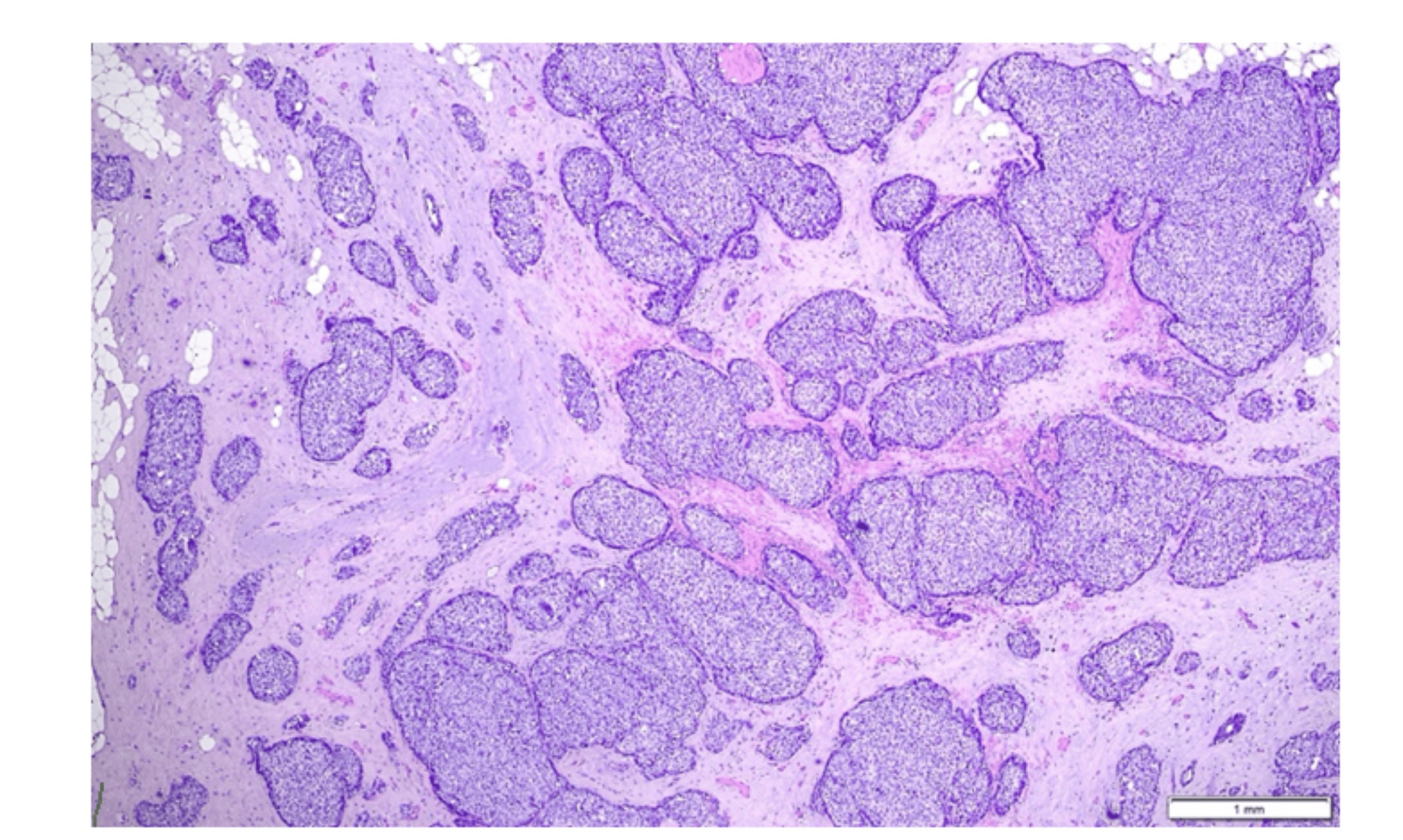
Solid aggregations or nests of tumor cells with pale granular cytoplasm and moderate nuclear pleomorphism (hematoxylin-eosin {H&E} staining; x4).

**Figure 3:**
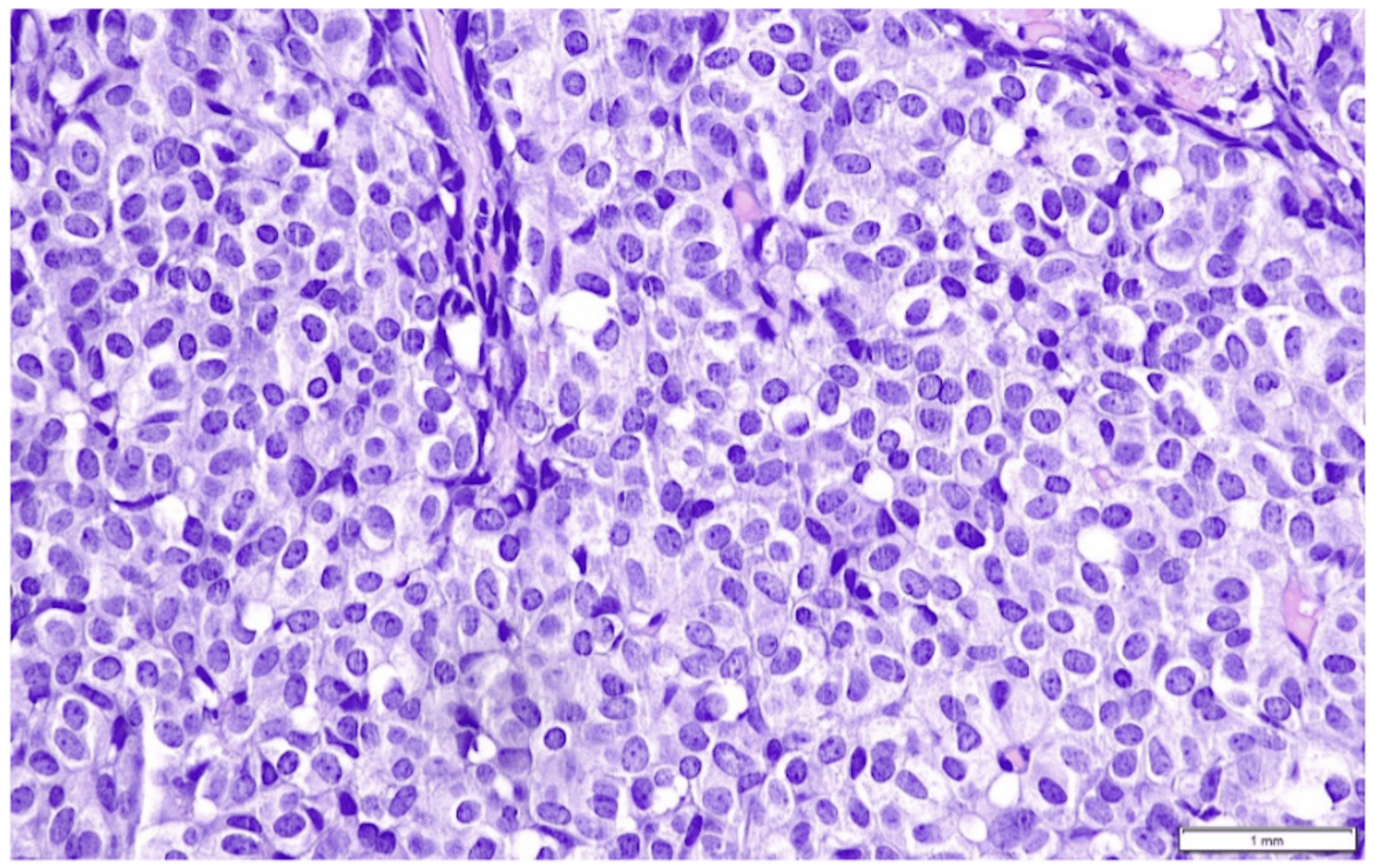
Tumor cells with pale granular cytoplasm and moderate nuclear pleomorphism (hematoxylin-eosin {H&E} staining; x40).

Histologically, the tumor is of intermediate differentiation (Nottingham Grade II), predominantly solid and nested with minor adenoid, cribriform, or trabecular patterns, composed of polygonal cells with pale granular cytoplasm, moderate nuclear pleomorphism, and low mitotic activity; no necrosis or mucin production is present. The tumor sections were processed using hematoxylin and eosin (H&E) staining to evaluate cellular morphology and tissue architecture (Figures 2, 3).

**Figure 4:**
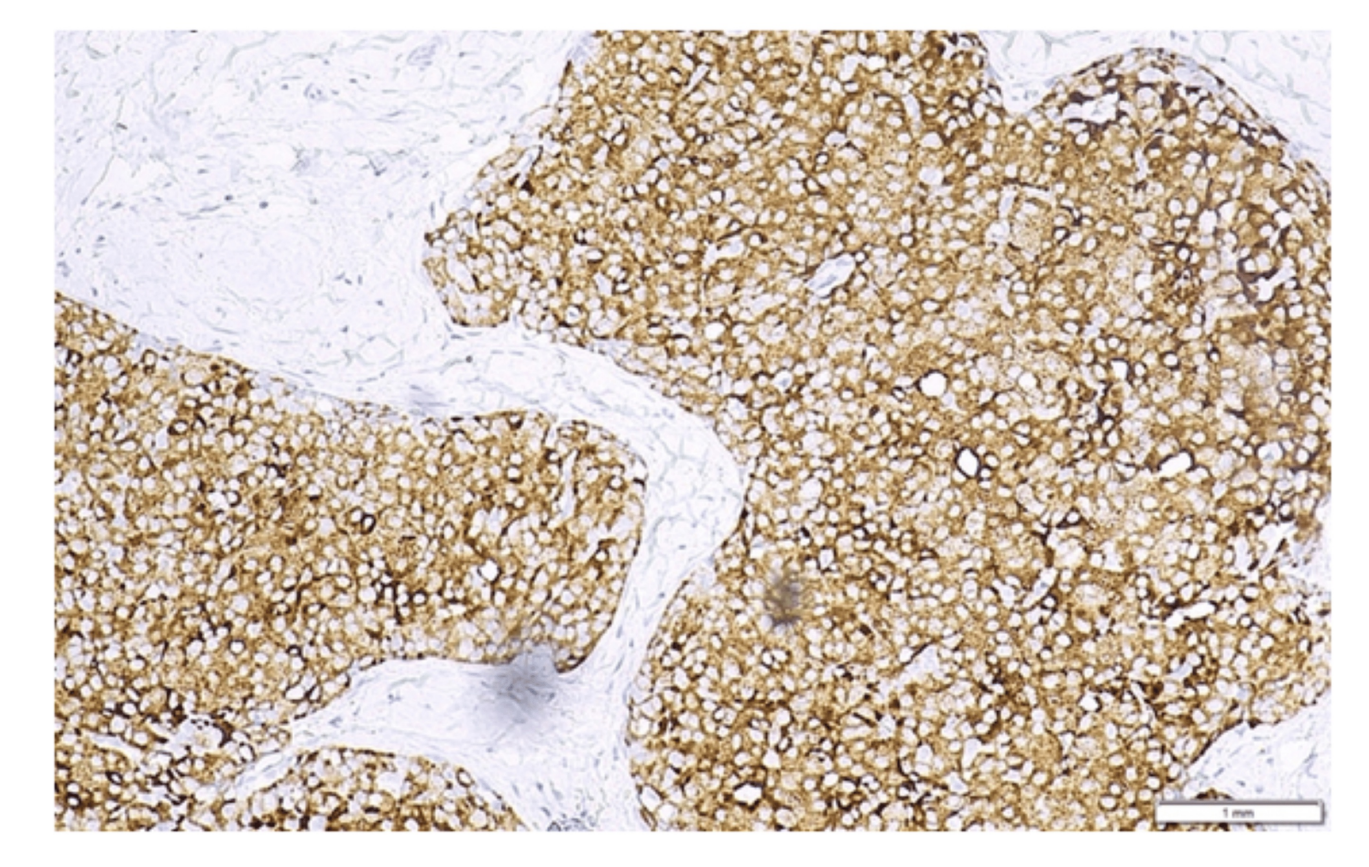
Synaptophysin-positive tumor cells showing diffuse expression (immunohistochemistry {IHC} staining; ×4).

**Figure 5:**
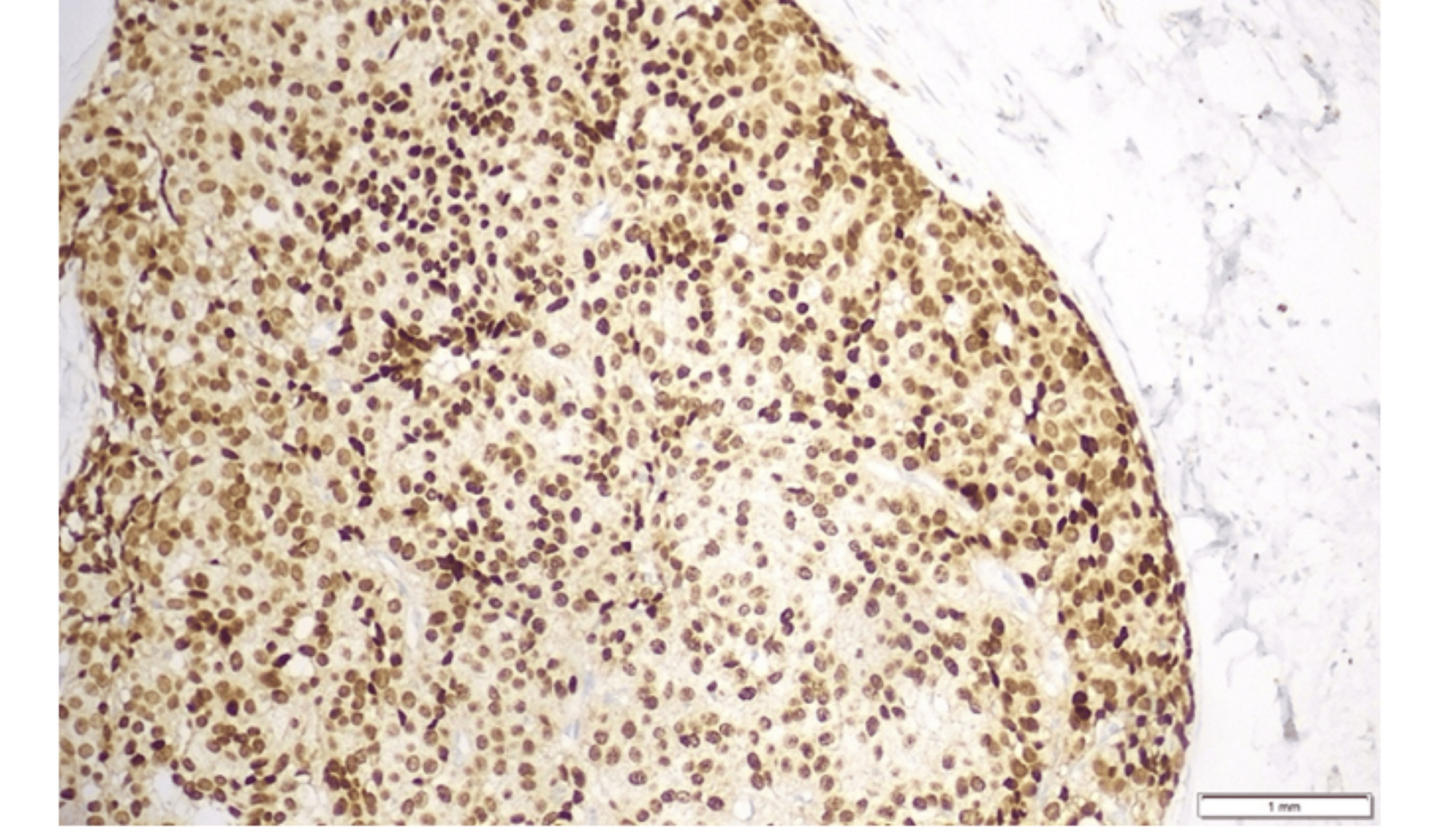
Tumor cells showing moderate to strong nuclear estrogen receptor positivity in ~95% of cells (immunohistochemistry {IHC}; ×10).

Immunohistochemistry revealed diffuse membranous E-cadherin positivity, diffuse nuclear GATA-3 positivity, negative TTF-1 and p63, diffuse synaptophysin positivity, focal chromogranin positivity, INSM-1 positivity in several cells, preserved YAP-1 in a few areas, RB-1 loss, and negative serotonin and CD56 (Figure 4). Somatostatin receptors SSTR2a and SSTR5 demonstrated moderate (++) positivity (Volante score 2, membrane expression in <50% of tumor cells). Surgical margins and overlying skin were free of tumor. Prognostic and predictive markers showed estrogen (ER) moderate to strong nuclear positivity in ~95% of tumor cells, PgR moderate to strong positivity in ~85% of tumor cells, low Ki-67 (~8%), and HER2 negativity (1+) (Figure 5). Given the above morphological and immunohistochemical findings, the diagnosis of a primary grade 2 well-differentiated tumor (NET G2) of the breast was made, tumor, node, metastasis (TNM) pathologic stage: pT1cNx (WHO/American Joint Committee on Cancer {AJCC}).

Follow-up and outcomes

The patient had been followed regularly for three years and three months (from December 2022 to April 2026) with clinical examination, imaging, and laboratory evaluation. No evidence of local recurrence or distant metastasis has been detected to date, and the patient remains in excellent general condition.

## Discussion

Across the literature, neuroendocrine carcinomas of the breast represent a very rare subtype of breast cancer, accounting for less than 0.1% of all breast cancers and less than 1% of all neuroendocrine tumors. Clinically, breast NETs most often present in postmenopausal women and are typically hormone receptor-positive and HER2-negative, aligning them with luminal-type breast cancers [6]. However, cases have also been reported in younger female patients and in men [7].

Notably, our patient was 88 years old, representing an older age than typically reported. No benign neuroendocrine tumors of the breast have ever been documented. One plausible explanation suggests that these types of tumors arise as a result of differentiation of invasive breast cancer, rather than from pre-existing endocrine cells of the breast [4]. Diagnosis is usually established by a pathologist after biopsy, since the clinical presentation and imaging findings are non-specific and similar to other types of breast cancer [3].

Breast NECs usually present as palpable and painless masses located in the retro-areolar area. Secondary symptoms, such as nipple retraction, fixation to adjacent structures, bloody nipple discharge, skin ulceration, and lymphadenopathy, have also been reported. In contrast, our case presented with a lesion located centrally and medial to the midline, without associated secondary symptoms.

Tumor size is generally small, with reported median sizes around 18 mm (range: 10-53 mm) [2]. Similarly, the tumor size in our patient was consistent with values reported in the literature. Occasionally, these types of tumors can secrete hormones, such as adrenocorticotropic hormone (ACTH), norepinephrine, or calcitonin, leading to paraneoplastic syndromes. In our case, laboratory evaluation, including ACTH and calcitonin levels, was performed and yielded negative results.

Radiological findings on mammography include an irregularly demarcated, round, and hyperdense mass. Breast ultrasound depicts a hypoechoic solid mass with cystic components, rich vascularity, and obscure borders, while MRI shows homogeneous, low signal intensity on T1-weighted images [5,8]. MRI findings are usually non-specific. In our case, imaging findings were highly suggestive of malignancy. Additionally, contrast-enhanced whole-body computed tomography (CT) showed no evidence of distant metastases.

It should be noted that PET-CT with 68 gallium-labeled somatostatin analogs can be used for well-differentiated tumors, while 18-fluorodeoxyglucose (FDG) PET-CT is reserved for breast NECs with poor differentiation or those with high metabolic activity. Most common metastatic sites include the bones, liver, lungs, brain, bone marrow, and pleura. Skin involvement has also been reported [9]. Both morphological features and neuroendocrine biomarkers guide the diagnostic process. However, imaging-guided (ultrasound, stereotactic guidance, or MRI) core-needle biopsy or surgical specimens are a prerequisite for definitive diagnosis, and this approach was likewise applied in our case [1,10,11].

In our case, the patient was diagnosed with grade 2 (G2) primary breast NET, intermediate-grade subgroup, characterized by moderate proliferative activity (mitotic count and Ki-67 index), bridging the biological spectrum between indolent NET G1 and aggressive NECs. NET G2 typically exhibits organoid, trabecular, or solid growth patterns, with uniform cells and finely granular chromatin, while immunohistochemical expression of synaptophysin and chromogranin A is required for confirming neuroendocrine differentiation [6].

From a clinical standpoint, breast NETs frequently demonstrate a luminal phenotype (ER/PR-positive, HER2-negative), supporting their biological relationship with conventional luminal-type breast carcinomas [12]. Available evidence suggests that well-differentiated NETs (G1-G2) may have a more favorable prognosis compared to NECs; however, outcome data remain inconsistent due to small cohorts and heterogeneity across studies [12,13].

A variety of differential diagnoses must be considered, particularly metastases from extra-mammary neuroendocrine tumors, which represent the most important condition to exclude. In our patient, thorough preoperative staging, including contrast-enhanced whole-body CT, bone scintigraphy, and laboratory evaluation with tumor markers, did not reveal any extra-mammary primary tumor.

NETs are characterized by a distinct metastatic pattern, most commonly involving the liver, followed by lymph nodes, bone, lungs, and peritoneum, while breast involvement remains rare [14]. Metastatic involvement of the breast from extra-mammary NETs accounts for approximately 1% of breast neoplasms [15]. Furthermore, recent data suggest that a significant proportion of patients with NECs may develop distant metastases within a few years of diagnosis, particularly in high-grade tumors [16].

Therefore, distinguishing between primary and metastatic neuroendocrine tumors of the breast is of critical importance, as it significantly impacts both therapeutic management and prognosis, while also highlighting the importance of comprehensive staging and long-term follow-up. In our case, the patient has been followed for more than three years with regular clinical, laboratory, and imaging evaluation, with no evidence of local recurrence or distant metastasis to date.

Regarding treatment, there are no specific guidelines for the therapy of breast NECs, owing to their extreme rarity and heterogeneity. Current evidence suggests that the management of neuroendocrine tumors of the breast largely follows treatment principles established for invasive breast carcinoma. Surgical resection remains the mainstay of treatment. Adjuvant radiotherapy following mastectomy is also an integral component of treatment, especially for tumors with good or moderate differentiation. Chemotherapy in the form of neoadjuvant or adjuvant treatment is usually reserved for locally advanced and metastatic disease or for tumors with a high risk of recurrence. Protocols that are usually implemented are similar to those that are used for classic, invasive breast carcinoma (e.g., epirubicin and cyclophosphamide) and small-cell lung cancer (e.g., carboplatin and etoposide). Notably, Gallo et al. mentioned a rare case of a HER-2 positive breast NEC, which was treated with trastuzumab, ultimately achieving a disease-free period of nine years [8]. It should be noted that the use of stomatostatin analogs (SSAs) for treatment has shown disappointing results. In hormone receptor-positive breast NECs, anti-hormonal therapy such as aromatase inhibitors has shown effectiveness in the form of adjuvant therapy. Last but not least, for tumors with proven somatostatin receptor positivity, peptide receptor radionuclide therapy (PRRT) can also be used, although it is reserved for specific cases as second-line treatment after failure of chemotherapy or as first-line treatment only in advanced disease [2,5-8,10,11,17].

The exact prognosis for breast NECs has yet to be established, as most studies do not take into consideration each and every specific subtype of breast NEC. Prognosis generally depends on the stage and histological grade of the tumor. Reportedly, five-year survival rates exceed 80% for all forms. Despite very little evidence, some studies suggest that neuroendocrine differentiation is an independent adverse prognostic factor in breast cancer, with higher local and distant recurrence rates. Small-cell carcinomas typically have the worst prognosis. However, it is unclear whether the degree of neuroendocrine marker expression has any prognostic significance [3,6,11]. Sali et al. noted that when they compared a group of patients with synaptophysin positivity only with a group that had both synaptophysin and chromogranin positivity, they found that the latter displayed lower Ki-67 index values and higher progesterone (PR) rates. Therefore, it is possible that this group may have a better prognosis [17].

In the present case, despite recommendations for more extensive surgical and adjuvant treatment, the patient underwent breast-conserving surgery alone and declined sentinel lymph node biopsy, radiotherapy, and chemotherapy. Importantly, she has remained disease-free during a follow-up period of three years and three months, with regular clinical, laboratory, and radiological surveillance. This case is noteworthy not only due to the rarity of primary neuroendocrine tumors of the breast but also because it highlights important clinical considerations in the management of elderly patients.

## Conclusions

This case highlights the rarity of primary neuroendocrine tumors of the breast and the importance of careful pathological evaluation. The advanced age of the patient, the comprehensive exclusion of an extra-mammary primary tumor, and the individualized treatment approach, with omission of adjuvant therapy, combined with a favorable long-term outcome, underscore the importance of tailored decision-making in this population.
